# A Novel and Expedient Approach to New Thiazoles, Thiazolo[3,2-*a*]pyridines, Dihydrothiophenes, and Hydrazones Incorporating Thieno[2,3-*b*]thiophene Moiety

**DOI:** 10.3390/ijms13045035

**Published:** 2012-04-23

**Authors:** Yahia Nasser Mabkhot, Assem Barakat, Abdullah Mohammad Al-Majid, Abdullah Saleh Alamary, Taleb T. Al-Nahary

**Affiliations:** 1 Department of Chemistry, Faculty of Science, King Saud University, P.O. Box 2455, Riyadh 11451, Saudi Arabia; E-Mails: amajid@ksu.edu.sa (A.M.A.-M.); alamary1401@yahoo.com (A.S.A.); 2 Department of Chemistry, Faculty of Applied Science, Thamar University, P.O. Box 87246, Yemen; E-Mail: alnaharyt@yahoo.com

**Keywords:** thiazolo[3,2-*a*]pyridine, dihydrothiphene, thieno[2,3-*b*]thiophene, symmetrical bis-heterocycle

## Abstract

This paper reports details about the synthesis of a series of novel functionalized symmetrical bis-heterocyclic compounds containing a thieno[2,3-*b*]thiophene motif. Bis-thiazole derivatives **2**, **3a-c** and thiazolo[3,2-*a*]pyridine derivatives **4a-c** are achieved. The hitherto unknown dihydrothiophene derivatives **6a-d** *via* bis-pyridimium salt **5** are obtained. Additionally, the novel hydrazonothieno[2,3-*b*]thiophene derivatives **10a-c** are obtained *via bis*-tosylacetylthieno[2,3-*b*]thiophene derivative **9**. All compounds are characterized by ^1^H-, ^13^C-NMR, GCMS, IR, and UV-vis spectrometry. These compounds represent a new class of sulfur and nitrogen containing heterocycles that should also be of interest as new materials.

## 1. Introduction

Thienothiophene derivatives have been developed for different purposes in the pharmaceutical field and have been tested as potential antitumor, antiviral, antibiotic, and antiglaucoma drugs, or as inhibitors of platelet aggregation [1–5]. We have been interested for some time in the chemical and biological properties of thienothiophene derivatives [6–12]. On the other hand thiophene derivatives represent a class of important and well-studied heterocycles [13,14]. Thiazoles and their derivatives have attracted continuing interest over the years because of their diverse biological activities [15,16]. They have found application in drug development for the treatment of allergies [15], hypertension, inflammation, schizophrenia, bacterial and HIV infections [17–21]. On the other hand substituted thiazolo pyridines represent an important class of annulated heterocycles with diverse types of pharmaceutical [22] and pesticide activity [23–28]. We have found that the *bis*-2-bromoacetylthieno[2,3-*b*]thiophene derivative **1**, is a versatile, readily accessible building block for the synthesis of several new heterocyclic compounds of biological potency.

## 2. Results and Discussion

**Scheme 1:**
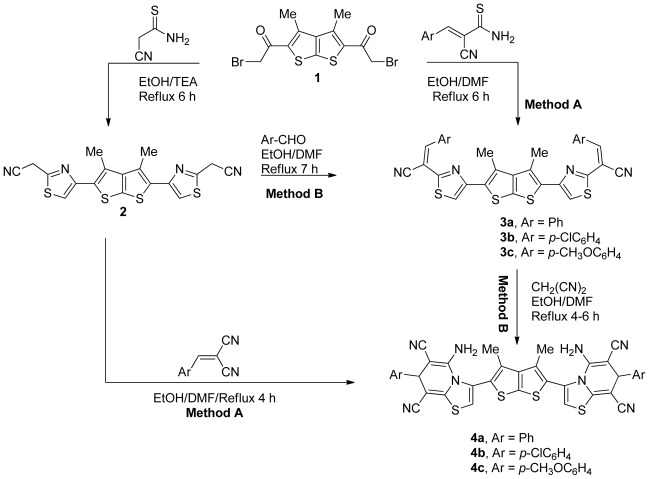
Synthesis of thiazolo[3,2-*a*]pyridine derivatives **4a-c**.

Refluxing of equimolar amounts of the *bis*-2-bromoacetylthieno[2,3-*b*]thiophene derivative **1** and cyanothioacetamide in ethanol and in the presence of a catalytic amount of TEA, afforded the corresponding *bis*-thiazole derivative **2**. The structure of the isolated cycloadduct was identified as 2,2′-(4,4′-(3,4′-dimethylthieno[2,3-*b*]thiophene-2,5-diyl)*bis*(thiazole-4,2-diyl))diacetonitrile (**2**) on the basis of its elemental analyses and spectral data. The IR spectrum of the reaction product exhibited absorption bands at 2259 cm^−1^ due to the nitrile group. The ^1^H-NMR spectrum of compound **2** revealed three singlets at *δ* 2.21, 3.79 and 7.80 due to methyl, methelene and CH thiazol protons, respectively. The reactivity of compound **2** towards some heterocyclic aldehydes was also investigated. Thus, the treatment of compound **2** with aldehyde derivatives in ethanol and in the presence of a catalytic amount of TEA afforded *bis*-thiazole derivatives **3a-c**. The ^1^H-NMR spectrum of **3a** showed three singlets at *δ* 2.28, 8.50 and 8.84 due to CH_3_, CH and CH thiazol protons respectively, in addition to an aromatic multiplet in the region 6.99–7.40. Compounds **3a-c** were alternatively obtained by the reaction of the treatment of *bis*-2-bromoacetylthieno[2,3-*b*]thiophene derivative **1** with 2-cyano-2-arylmethylene-thioacetamide derivatives in ethanol/DMF (Scheme 1). Treatment of the *bis*-thiazole derivatives **3a-c** with malononitrile afforded the corresponding thiazolo pyridine derivatives **4a-c** (Scheme 1). Compounds **4a-c** were alternatively obtained by reaction of the treatment of *bis*-thiazole derivative **2** with 2-cyano-2-arylmethylenethioacetamide derivatives in ethanol/DMF (Scheme 1). On the other hand, compounds **4a-c** underwent a thermal intramolecular cyclization reaction *via* an initial Michael type adduct. The IR spectrum of compound **4b**, taken as a typical example of the prepared series, revealed absorption bands at 2241, 2193, and 3383–3320 cm^−1^ corresponding to two nitrile and amino functions, respectively. Its ^1^H-NMR spectrum showed signals at *δ* 2.31, 4.57, 4.72, and 9.23, due to CH_3_, CH_2_, NH_2_ and CH thiazol protons respectively, in addition to an aromatic multiplet in the region 6.99–7.40. An aromatic multiplet in the region *δ* 7.49–7.60 was also found. Its mass spectrum revealed a molecular ion peak at *m/z* 789.

**Scheme 2:**
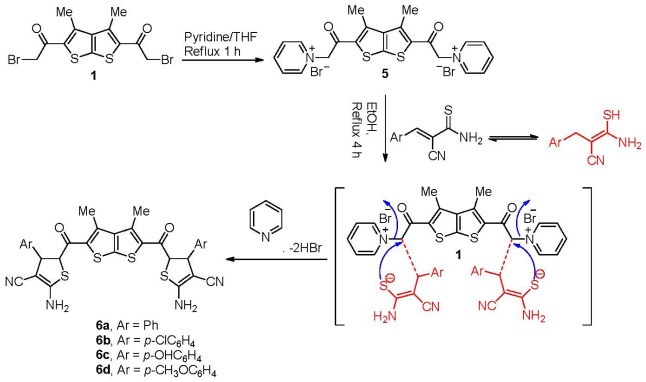
Synthesis of dihydrothiophene derivatives **6a-d**.

Our study was extended to include the synthesis of new bis-dihydrothiophene derivatives **6a-d**. Thus, the *bis*-2-bromoacetylthieno[2,3-*b*]thiophene **1** was refluxed in a mixture of absolute ethanol, pyridine and THF for 1 h to give a single product of *bis*-pyridimium salt **5** as examined by TLC. Elemental analyses and mass spectrum analysis of the isolated product were completely in agreement with the molecular formula C_22_H_20_Br_2_N_2_O_2_S_2_. The structure of the product is assumed to be **5** according to the rationale outlined in Scheme 2 in 95% yield. The compound reacts with (*E*)-3-amino-2-benzyl-3-mercaptoacrylonitrile which is in resonating structure with (*E*)-2-cyano-3-phenylprop-2-enethioamide in refluxing ethanol which undergoes intramolecular cyclization to give compound **6a** in 86% yield. The ^1^H-NMR of compound **6a** was free of pyridine protons and exhibited two characteristic doublet signal at 4.40, 4.70 pmm integrated for 2H (for the C–H proton of the dihydrothiophene moiety). In addition, one broad signal at 4.19 ppm was integrated for 2H proton (for the NH_2_). Furthermore, CN and NH absorption appeared at 2187, 3450 cm^−1^, respectively, in the IR.

It is noteworthy to mention here that the *bis*-dihydrothiophene derivatives **6b-d** with different moieties were also prepared from *bis*-pyridimium salt **5** and the corresponding aryl-mercaptoacrylonitrile derivatives but in very good yield, as depicted in Scheme 2.

**Scheme 3:**
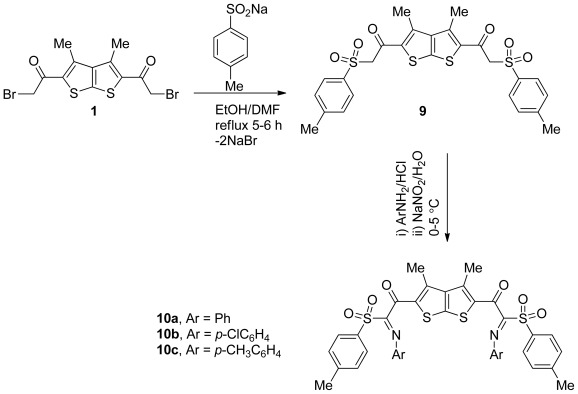
Synthesis of hydrazonothieno[2,3-*b*]thiophene derivatives **10a**–**c**.

There has been continuous interest in the synthesis of a new hydrazono system containing sulfone moiety because it holds considerable interest relative to the preparation of organic intermediates and physiologically active compounds. Thus, when the *bis*-2-bromoacetylthieno[2,3-b]thiophene 1 was treated with sodium 4-methylbenzenesulfinate in a mixture of absolute ethanol and DMF under reflux for 4 h, it afforded a white crystalline product, namely the *bis*-tosylacetylthieno[2,3-b]thiophene derivative 9 in a 97% yield, and its use as key intermediate for the synthesis of a wide variety of *bis*-(hydrazones) derivative 10 is shown in Scheme 3.

When *bis*-tosylacetylthieno[2,3-*b*]thiophene derivative **9** were allowed to react with benzenediazoniumchloride, which had been prepared *in situ* from the corresponding aniline in hydrochloric acid with aqueous sodium nitrite in dioxane at 0–5 °C, it resulted in a single product as examined by TLC. Elemental analyses and mass spectrum analysis of the isolated product were completely in agreement with the molecular formula C_40_H_32_O_6_S_4_. The structure of the product is assumed to be **10a** according to the rationale outlined in Scheme 3 in a 76% yield. The structure of compound **10a** was substantiated from its elemental and spectral analyses. Its IR spectrum showed the presence of an absorption band characteristic for NH as well as the presence of C=N absorption at 3217, and 1627 cm^−1^, respectively. The fact that the ^1^H NMR of compound **10a** was free of tosylacetyl protons in the ^1^H NMR spectrum strongly supported this assignment.

Finally, having now available the new *bis*-tosylacetylthieno[2,3-*b*]thiophene derivative **9** prompted us to study its synthetic utility as a key intermediate for novel symmetrical *bis*-(hydrazono) heterocycles **10b**,**c**. Following the same methodology as described for **10a** resulted in the formation of the *bis*-(hydrazone) derivatives **10b** and **10c** in 81% and 77% yield, respectively, and as depicted in Scheme 3. The structures of compounds **10b**,**c** were inferred from different spectroscopic and analytical data.

## 3. Experimental Section

All melting points were measured on a Gallenkamp melting point apparatus. IR spectra were measured as KBr pellets on a Pye-Unicam SP 3–300 spectrophotometer. The NMR spectra were recorded on a Varian Mercury VX-400 NMR spectrometer. ^1^H-NMR and ^13^C-NMR (400 MHz) were run in dimethylsulphoxide (DMSO-*d*_6_). Chemical shifts were related to that of the solvent. Mass spectra were recorded on a Shimadzu GCMS-QP 1000 EX mass spectrometer at 70 eV. Elemental analyses was carried out on an Elementar Vario EL analyzer.

### 3.1. 2,2′-(4,4′-(3,4′-Dimethylthieno[2,3-*b*]thiophene-2,5-diyl)*Bis*(thiazole-4,2 diyl))diacetonitrile *(***2***)*

To a solution of **1** (0.41 g, 1 mmol, 1.0 equiv) in absolute ethanol (20 mL, 99.9%), 2-cyanoethanethioamide (0.20 g, 2.0 mmol, 2.0 equiv) and TEA (triethylamine) were added (few drops) and the resulting mixture was refluxed for 6 h. The solution was allowed to cool to room temperature. The formed solid product was filtered off and recrystallized from EtOH/DMF to afford the compound **2** as black needle crystals; Yield (88%); m.p. 270–272 °C; IR *ν*_max_ (KBr) 2259 (CN), 1529 (C=N) cm^−1; 1^H-NMR (400 MHz, DMSO-*d*_6_) (ppm): 2.21 (s, 6H, CH_3_), 3.79 (s, 4H, CH_2_), 7.80 (s, 2H, Thiazol); ^13^C-NMR: *δ* 14.8, 22.0, 117.4, 116.8, 128.8, 159.3, 134.3, 136.0, 148.1, 148.8; MS *m/z* (%): 412 (M**^+^**, 100); Anal. for C_18_H_12_N_4_S_4_ (412.05) calcd; C, 52.40; H, 2.93; N, 13.58; S, 31.09. Found: C, 52.10; H, 2.71; N, 13.28; S, 31.42.

### 3.2. General Procedure for the Synthesis of Compounds **3a-c** (GP1)

#### 3.2.1. 4,4′-(3,4-Dimethylthieno[2,3-*b*]thiophene-2,5-diyl)*bis*(thiazole-4,2-Diyl))*bis* (3-aryle acrylonitrile) (**3a**–**c**)

**Method A:** To a solution of **1** (0.41 g, 1 mmol, 1.0 equiv.) in mixture of absolute ethanol (20 mL, 99.9%) and DMF (5 mL), 3-aryle-2-cyanoprop-2-enethioamide (2.0 mmol, 2.0 equiv.) was added, and the reaction mixture was then heated under reflux for 6 h. The solution was allowed to cool to room temperature. The solid product was collected by filtration and recrystallized from EtOH/DMF to afford the compound **3a**–**c**.

**Method B:** To a solution of **2** (0.41 g, 1 mmol, 1.0 equiv) in mixture of absolute ethanol (20 mL, 99.9%) and DMF (5 mL), aromatic aldehyde derivatives (2 mmol, 2.0 equiv) were added, the reaction mixture was then heated under reflux for 6–7 h. The solution was allowed to cool to room temperature. The solid product was collected by filtration and recrystallized from EtOH/DMF to afford the compound **3a**–**c**.

#### 3.2.2. 2,2′-(4,4′-(3,4-Dimethylthieno[2,3-*b*]thiophene-2,5- diyl)*bis*(triazole -4,2-diyl))*bis* (3-phenylacrylonitrile) (**3a**)

**3a** was prepared according to method A or method B, dark yellow crystals; yield (81**^a^**, 67**^b^** %); m.p. 300–302 °C; IR *ν*_max_ (KBr) 2214 (CN), 1591 (C=N) cm^−1; 1^H-NMR (400 MHz, DMSO-*d*_6_) (ppm): 2.28 (s, 6H, CH_3_), 6.99–7.40 (m, 10H, ArH’s), 8.50 (s, 2H, Ar–CH=C), 8.84 (s, 2H, Thiazol); ^13^C-NMR: *δ* 15.5, 118.0, 113.2, 135.9, 164.5, 106.1, 154.0, 124.0, 125.2, 128.0, 132.0, 138.5, 141.4, 147.6, 148.2; MS *m/z* (%): 588 (M**^+^**, 100); Anal. for C_32_H_20_N_4_S_4_ (588.79) calcd; C, 65.28; H, 3.42; N, 9.52; S, 21.78. Found: C, 65.06; H, 3.18; N, 9.23; S, 21.12.

#### 3.2.3. 2,2′-(4,4′-(3,4-Dimethylthieno[2,3-*b*]thiophene-2,5-diyl)*bis*(thiazole-4,2-Diyl))*bis* (3-(4-chlorophenyl)acrylonitrile (**3b**)

**3b** was prepared according to method A or method B, brown needle crystals, yield (75**^a^**, 48**^b^** %); m.p. >320 °C; IR ν_max_ (KBr) 2119 (CN), 1570 (C=N) cm^−1; 1^H-NMR (400 MHz, DMSO-*d*_6_) (ppm): 2.36 (s, 6H, CH_3_), 6.44 (d, 2H, *J* = 8.4 Hz, ArH’s), (d, 2H, *J* = 8.4 Hz, ArH’s), 8.48 (s, 2H, Ar–CH=C), 8.82 (s, 2H, Thiazol), ^13^C-NMR: *δ* 14.1, 117.7, 111.1, 149.5, 162.3, 104.8, 153.2, 122.4, 125.8, 127.3, 131.2, 134.2, 138.1, 142.5, 147.9; MS *m/z* (%): 658 (M^+^+2, 62); Anal. for C_32_H_18_N_4_S_4_ Cl_2_ (657.68) calcd; C, 58.44; H, 2.76; N, 8.52; S, 19.50. Found: C, 58.14; H, 2.46; N, 8.82; S, 19.20.

#### 3.2.4. 2,2′-(4,4′-(3,4-Dimethylthieno[2,3-*b*]thiophene-2,5-diyl)*bis*(thiazole-4,2-diyl))*bis* (3-(4-methoxyphenyl)acrylonitrile (**3c**)

**3c** was prepared from according to method A or method B (*GP1*), dark brown powder crystals; yield (88**^a^**, 55**^b^** %); m.p. 282–284 °C; IR *ν*_max_ (KBr) 2188 (CN), 1579 (C=N) cm^−1; 1^H-NMR (400 MHz, DMSO-*d*_6_) (ppm): 2.36 (s, 6H, CH_3_), 3.86 (s, 6H, OCH_3_), 6.57 (d, 2H, *J* = 8.4 Hz, ArH’s), 7.21 (d, 2H, *J* = 8.4 ArH’s) 8.43 (s, 2H, Ar–CH=C), 8.87 (s, 2H, Thiazol); ^13^C-NMR: *δ* 14.6, 116.4, 112.45, 148.7, 164.2, 102.1, 155.8, 123.4, 125.1, 128.8, 129.3, 55.4, 133.8, 137.6, 141.9, 146.3; MS *m/z* (%): 648 (M^+^, 100); Anal. for C_32_H_18_N_4_S_4_ Cl_2_ (648.84) calcd; C, 62.94; H, 3.73; N, 8.63; S, 19.77. Found: C, 62.64; H, 3.43; N, 8.93; S, 19.47.

### 3.3. General Procedure for the Synthesis of Compounds **4a-c** (GP2)

#### 3.3.1. 3,3′-(3,4-Dimethylthieno[2,3-*b*]thiophene-2,5-diyl)*bis*(5-amino-7-aryle-7H-thiazolo[3,2-*a*]pyridine-6,8-dicarbonitrile) (**4a-c**)

**Method A:** To a solution of **2** (0.41 g, 1 mmol, 1.0 equiv) in mixture of absolute ethanol (20 mL, 99.9%) and DMF (5 mL), 2-arylidenemalo nonitril derivatives (2 mmol, 2.0 equiv) were added, the reaction mixture was then heated under reflux for 4 h. The solution was allowed to cool to room temperature. The solid product was collected by filtration and recrystallized from EtOH/DMF to afford the compound **4a-c**.

**Method B:** To a solution of **3a-c** (1.0 mmol, 1.0 equiv) in mixture of absolute ethanol (20 mL, 99.9%) and DMF (5 mL), malononitrile (0.13 mL, 2.0 mmol, 2 equiv) was added, the reaction mixture was then heated under reflux for 4–6 h. The solution was allowed to cool to room temperature. The solid product was collected by filtration and recrystallized from EtOH/DMF to afford the compound **4a-c**.

#### 3.3.2. 3,3′-(3,4-Dimethylthieno[2,3-*b*]thiophene-2,5-diyl)*bis*(5-amino-7-phenyl-7H-thiazolo [3,2-a]pyridine-6,8-dicarbonitrile (**4a**)

**4a** was prepared according to method A or method B (GP2), black light needle crystals; yield (73**^a^**, 56**^b^** %); m.p. >302 °C; IR *ν*_max_ (KBr) 3444–3361(NH_2_ ), 2202, 2188.8 (4CN) cm^−1; 1^H-NMR (400 MHz, DMSO-*d*_6_) (ppm): 2.21 (s, 6H, CH_3_), 4.37 (br, 4H, NH_2_), 5.02 (s, 2H, pyridine), 7.04–7.70 (m, 10H, ArH’s), 9.08 (s, 2H, Thiazol); ^13^C-NMR: *δ* 13.8, 115.7, 116.9, 112.1, 153.9, 155.0, 33.0, 56.4, 71.5, 159.8, 123.0, 126.0, 128.9, 134.3, 141.5, 141.9, 148.1, 148.8; MS *m/z* (%):720 (M^+^, 100); Anal. for C_38_H_24_N_8_S (720.91) calcd; C, 63.31; H, 3.36; N, 15.54; S, 17.79. Found: C, 63.01; H, 3.06; N, 15.24; S, 17.49.

#### 3.3.3. 3,3′-(3,4-Dimethylthieno[2,3-*b*]thiophene-2,5-diyl)*bis*(5-amino-7-(4-chlorophenyl)-7Hthiazolo[ 3,2-*a*]pyridine-6,8-dicarbonitrile (**4b**)

**4b** was prepared according to method A or method B (GP2), greenish yellow scale crystals; yield (85**^a^**, 62**^b^** %); m.p. >302 °C; IR *ν*_max_ (KBr) 3383–3320 (NH_2_ ), 2241, 2193.2 (4CN) cm^−1; 1^H-NMR (400 MHz, DMSO-*d*_6_) (ppm): 2.31 (s, 6H, CH_3_), 4.57 (br,4H, NH_2_), 4.72 (s, 2H, pyridine), 7.17 (d, 2H, *J* = 8.8 Hz, ArH’s), 7.65 (d, 2H, *J* = 8.8 Hz, ArH’s), 9.23 (s, 2H, Thiazol); ^13^C-NMR: *δ* 15.2, 117.6, 118.4, 114.6, 152.5, 155.2, 32.3, 58.2, 73.8, 158.5, 122.7, 125.1, 127.6, 132.3, 142.7, 142.8, 147.5, 148.1; MS *m/z* (%):790 (M^+^, 18); Anal. for C_38_H_22_N_8_S_4_ Cl_2_ calcd; C, 57.79; H, 2.81; N, 14.19; S, 16.24. Found: C, 57.49; H, 2.51; N, 13.97; S, 16.54.

#### 3.3.4. 3,3′-(3,4-Dimethylthieno[2,3-*b*]thiophene-2,5-diyl)*bis*(5-amino-7-(4-methoxyphenyl)-7Hthiazolo[ 3,2-a]pyridine-6,8-dicarbonitrile) (**4c**)

**4c** was prepared according to method A or method B (GP2), black shiny powder crystals; yield (68**^a^**, 44**^b^** %); m.p. >302 °C; IR ν_max_ (KBr) 3410–3315(NH_2_ ), 2288, 2245 (4CN) cm^−1; 1^H-NMR (400 MHz, DMSO-*d*_6_) (ppm): 2.28 (s, 6H, CH_3_), 3.85 (s, 6H, OCH_3_), 4.18 (br,4H, NH_2_), 5.23 (s, 2H, pyridine), 6.87 (d, 2H, *J* = 8.8 Hz, ArH’s), 7.12 (d, 2H, *J* = 8.8 Hz, ArH’s), 9.15 (s, 2H, Thiazol); ^13^C-NMR: *δ* 13.4, 117.48, 119.11, 111.1, 154.3, 156.09, 34.0, 57.1, 75.1, 159.6, 122.7, 125.1, 127.6, 132.3, 51.3, 141.2, 142.3, 148.2, 148.4; MS *m/z* (%): 780 (M^+^, 100); Anal. for C_40_H_28_N_8_O_2_S_4_ calcd; C, 61.52; H, 3.61; N, 14.35; S, 16.42. Found: C, 61.22; H, 3.31; N, 14.65; S, 16.20.

### 3.4. 1,1′-(2,2′-(3,4-Dimethylthieno[2,3-*b*]thiophene-2,5-diyl)*bis*(2-oxoethane-2,1-diyl))dipyridinium Bromide *(***5***)*

To a solution of **1** (0.41 g, 1.0 mmol, 1.0 equiv) in a mixture of absolute ethanol (20 mL, 99.9%) and THF (5 mL), pyridine (0.16 mL, 2 mmol, 2.0 equiv) was added, the reaction mixture was then heated under reflux for 1 h. The solution was allowed to cool to room temperature. The solid product was collected by filtration and recrystallized from EtOH/DMF to afford the compound **5**. White needles crystals; yield (95 %); m.p. 265–267 °C; IR *ν*_max_ (KBr) 1636 (C=O ), 1580 (C=N) cm^−1; 1^H-NMR (400 MHz, DMSO-*d*_6_) (ppm): 2.14 (s, 6H, CH_3_), 4.81 (s,4H, CH_2_), 8.27–9.06 (m, 10H, pyridine); ^13^C-NMR: *δ* 15.6, 67.6, 128.1, 128.9, 140.8, 141.6, 146.7, 146.9, 147.5, 147.9; MS *m/z* (%): 569 (M^+^ +4, 18); Anal. for C_22_H_20_Br_2_N_2_O_2_S_2_ calcd; C, 46.49; H, 3.55; N, 4.63 S, 11.28. Found: C, 46.19; H, 3.25; N, 4.63; S, 11.08.

### 3.5. General Procedure for the Synthesis of Compounds **6a-d** (GP3)

#### 3.5.1. 5,5′-(3,4-Dimethylthieno[2,3-*b*]thiophene-2,5-dicarbonyl)*bis*(2-amino-4-aryl-4, 5-dihydrothiophene-3-carbonitrile) (**6a-d**)

To a solution of **5** (0.57 gm, 1.0 mmol, 1.0 equiv) in mixture of absolute ethanol (20 mL, 99.9%) and DMF (5 mL), 2-cyano-3-aryle prop-2-enethioamide derivatives (2 mmol, 2.0 equiv) were added; the reaction mixture was then heated under reflux for 4 h. The solution was allowed to cool to room temperature. The solid product was collected by filtration and recrystallized from EtOH/DMF to afford the compound **6a-d**.

#### 3.5.2. 4,4′-5,5′(3,4-Dimethylthieno[2,3-*b*]thiophene-2,5-dicarbonyl)*bis*(2-amino-4-phenyl-4, 5-dihydrothiphene-3-corbonitrile (**6a**)

**6a** was prepared according to (GP3), red light powder crystals; yield (86%); m.p. 300–302 °C; IR *ν*_max_ (KBr) 3450–3348(NH_2_), 1651 (C=O) cm^−1; 1^H-NMR (400 MHz, DMSO-*d*_6_) (ppm): 2.25 (s, 6H, CH_3_), 4.19 (br, 4H, NH_2_), 4.40 (d, 2H, *J* = 4.80 Hz, dihydrothiophene), 4.70 (d, 2H, *J* = 4.20 Hz, dihydrothiophene), 7.26–7.39 (m, 10H, ArH’s); ^13^C-NMR: δ 14.8, 54.0, 55.2, 72.3, 159.3, 116.8, 123.6, 124.06, 128.8, 134.3, 136.0, 142.0, 148.1, 148.8, 191.0; MS *m/z* (%):624 (M^+^, 29); Anal. for C_32_H_24_N_4_O_2_S_4_ calcd; C, 61.51; H, 3.87; N, 8.97; S, 20.53. Found: C, 61.21; H, 3.57; N, 8.67; S, 20.83.

#### 3.5.3. 4,4′-5,5′(3,4-Dimethylthieno[2,3-*b*]thiophene-2,5-dicarbonyl)*bis*(2-amino-4-(4-chlorophenyl)-4,5-dihydrothiphene-3-corbonitrile (**6b**)

**6b** was prepared according to (GP3), dark brown powder crystals; yield (78%); m.p. >320 °C; IR *ν*_max_ (KBr) 3438–3320 (NH_2_), 1686 (C=O) cm^−1; 1^H-NMR (400 MHz, DMSO-*d*_6_) (ppm): 2.37 (s, 6H, CH_3_), 4.42 (br, 4H, NH_2_), 4.39 (d, 2H, *J* = 4.80 Hz, dihydrothiophene), 4.82 (d, 2H *J* = 4.20 Hz, dihydrothiophene), 7.12 (d, 2H, *J* = 8.0 Hz, ArH’s), 7.42 (d, 2H, *J* = 8.0 Hz, ArH’s); ^13^C-NMR: *δ* 15.6, 54.6, 56.1, 73.6, 161.1, 117.2, 122.3, 126.1, 133.8, 134.4, 139.6, 146.8, 147.1, 148.4, 188.7; MS *m/z* (%):694 (M^+^ +2, 19); Anal. for C_32_H_22_Cl_2_N_4_O_2_S_4_ calcd; C, 55.40; H, 3.20; N, 8.08; S, 18.49. Found: C, 55.10; H, 3.50; N, 7.88; S, 18.19.

#### 3.5.4. 4,4′-5,5′(3,4-Dimethylthieno[2,3-*b*]thiophene-2,5-dicarbonyl)*bis*(2-amino-4-(4-hydroxyphenyl)-4,5-dihydrothiphene-3-corbonitrile (**6c**)

**6c** was prepared according to (GP3), red blessed scale crystals; yield (79%); m.p. >320 °C; IR *ν*_max_ (KBr) 3420–3382(NH_2_), 1633 (C=O) cm^−1; 1^H-NMR (400 MHz, DMSO-*d*_6_) (ppm): 2.81 (s, 6H, CH_3_), 5.02 (br, 4H, NH_2_), 4.30 (d, 2H, *J* = 4.8 Hz, dihydrothiophene), 4.79 (d, 2H, *J*= 4.2 Hz, dihydrothiophene), 7.26 (d, 2H, *J* = 8.0 Hz, ArH’s), 7.57 (d, 2H, *J* = 8.0 Hz, ArH’s), 11.32 (br, 2H, OH); ^13^C-NMR: *δ* 15.4, 55.6, 56.6, 74.0, 152.7, 116.2, 123.2, 127.7, 134.5, 136.8, 142.2, 146.3, 148.1, 148.8, 190.4; MS *m/z* (%): 656 (M^+^, 100); Anal. for C_32_H_24_N_4_O_4_S_4_ calcd; C, 58.52; H, 3.68; N, 8.53; S, 19.53. Found: C, 58.22; H, 3.38; N, 8.23; S, 19.23.

#### 3.5.5. 4,4′-5,5′(3,4-Dimethylthieno[2,3-*b*]thiophene-2,5-dicarbonyl)*bis*(2-amino-4-(4-methoxyphenyl)-4,5-dihydrothiphene-3-corbonitrile (**6d**)

**6c** was prepared according to (GP3), dark yellow powder crystals; yield (81%); m.p. >320 °C; IR *ν*_max_ (KBr) 3433–3364 (NH_2_), 1710 (C=O) cm^−1; 1^H-NMR (400 MHz, DMSO-*d*_6_) (ppm): 2.33 (s, 6H, CH_3_), 3.81 (s, 6H, OCH_3_), 4.88 (br, 4H, NH_2_), 4.32 (d, 2H, *J* = 4.8 Hz, dihydrothiophene), 4.66 (d, 2H *J* = 4.2 Hz, dihydrothiophene), 6.85 (d, 2H, *J* = 8.0 Hz, ArH’s), 7.31 (d, 2H, *J* = 8.0 Hz, ArH’s); ^13^C-NMR: *δ* 15.3, 52.3, 56.2, 57.3, 74.24, 158.50, 115.9, 122.7, 128.5, 134.4, 135.8, 143.9, 147.0, 148.1, 148.5, 192.1; MS *m/z* (%):684 (M^+^, 100); Anal. for C_34_H_28_N_4_O_4_S_4_ calcd; C, 59.63; H, 4.12; N, 8.18; S, 18.73. Found: C, 59.33; H, 4.42; N, 8.48; S, 18.43.

### 3.6. 1,1′-(3,4-Dimethyl-5-(2-tosylacetyl)thieno[2,3-*b*]thiophen-2-yl)-2-tosylethanone *(***9***)*

To a solution of **8** (0.41 gm, 1.0 mmol, 1.0 equiv) in mixture of absolute ethanol (20 mL, 99.9%) and DMF (5 mL), sodium 4-methylbenzenesulfinate (0.35 g, 2 mmol, 2.0 equiv) was added; the reaction mixture was then heated under reflux for 4 h. The solution was allowed to cool to room temperature. The solid product was collected by filtration and recrystallized from EtOH/DMF to afford the compound **9**. White cubes crystals; yield (97%); m.p. 263–265 °C; IR *ν*_max_ (KBr) 2980 (CH_2_), 1653 (C=O) cm^−1; 1^H-NMR (40 0 MHz, DMSO-*d*_6_) (ppm): 1.50 & 2.73 (s, 12H, CH_3_), 3.90 (s, 4H, Methylene), 6.93 (d, 2H, *J* = 7.3 Hz, ArH’s), 6.99 (d, 2H, *J* = 7.3 Hz, ArH’s); ^13^C-NMR: *δ* 15.6, 20.6, 69.2, 122.0, 122.5, 128.2, 132.0, 138.6, 141.5, 146.0, 147.7, 190.1; MS *m/z* (%): 560 (M^+^, 52); Anal. for C_26_H_24_O_6_S_4_ calcd; C, 55.69; H, 4.31; S, 22.87. Found C, 55.39; H, 4.61; S, 22.67.

### 3.7. General Procedure for the Synthesis of Compounds **10a-c** (GP4)

#### 3.7.1. 2-(2-Arylhydrazono)-1-(5-((*Z*)-2-(2-chlorohydrazono)-2-tosylacetyl)-3,4-dimethylthieno [2,3-*b*]thiophen-2-yl)-2-tosylethanone (**10a**–**c**)

A stirred soln. of **9** (0.5 mmol, 0.28 g) in ethanol (15 mL) was cooled in an ice bath at 0–5 °C, and then a soln. of the benzenediazonium chloride [freshly prepared by diazotizing aniline (1 mmol) in HCl (0.28 mL) with NaNO_2_ (2 mmol) in H_2_O (4 mL)] was added drop-wise over a period of 20 min. The reaction mixture was kept in a refrigerator overnight. The solid product was collected by filtration, and recrystallized from Et-OH/DMF. Thus pure crystals of the desired compounds **10a**–**c** were obtained in a good yield that ranged from 76 to 81%.

#### 3.7.2. 1-(3,4-Dimethyl-5-2-(2-phenylhydrazono)-2-tosylacetyl)thieno[2,3-*b*]thiophen-2-yl)-2-(2- phenylhydrazono)-2-tosylethanone (**10a**)

**10a** was prepared according to (GP4), red light powder crystals; yield (76%); m.p.295–297 °C; IR *ν*_max_ (KBr) 3217 (NH), 1669 (C=O), 1595 (C=N) cm^−1; 1^H-NMR (400 MHz, DMSO-*d*_6_) (ppm): 2.14, 2.79 (s, 12H, CH_3_), 7.48–7.58 (m, 10H, aromatic), 7.83 (d, 2H, *J* = 7.3 Hz, ArH’s), 7.85 (d, 2H, *J* = 7.3 Hz, ArH’s), 8.25 (br, 2H, NH); ^13^C-NMR: *δ* 15.5, 21.6, 119.8, 119.8, 120.0, 120.2, 128.7, 130.2, 135.1, 138.0, 141.5, 145.3, 148.1, 148.6, 169.0, 185.3; MS *m/z* (%):768 (M^+^, 19); Anal. for C_38_H_32_N_4_O_6_S_4_ calcd; C, 59.35; H, 4.19; N, 7.29; S, 16.68. Found: C, 59.05; H, 4.49; N, 6.99; S, 16.48.

#### 3.7.3. 2-(2-(4-Chlorophenyl) hydrazono)-1-(5-2-(2-(4-chlorophenyl) hydrazono)-2-tosylacetyl)-3, 4-dimethylthieno[2,3-*b*]thiophen-2-yl)-2-tosylethanone (**10b**)

**10b** was prepared according to (GP4), yellow powder crystals; yield (81%); m.p. >320 °C; IR *ν*_max_ (KBr) 3288 (NH), 1720 (C=O), 1565 (C=N) cm^−1; 1^H-NMR (400 MHz, DMSO-*d*_6_) *δ* (ppm): 2.10, 2.86 (s, 12H, CH_3_), 7.19 (d, 2H, *J* = 8.8Hz, ArH’s), 7.28 (d, 2H, *J* = 8.8 Hz, ArH’s), 7.67 (d, 2H, *J* = 7.3 Hz, ArH’s), 7.80 (d, 2H, *J* = 7.3 Hz, ArH’s), 12.8 (br, 2H, NH); ^13^C-NMR: *δ* 14.8, 23.5, 121.2, 123.0, 123.9, 124.0, 126.3, 128.8, 132.4, 136.5, 140.9, 147.5, 147.7, 154.3, 165.2, 190.1; MS *m/z* (%): 838 (M^+^, 39); Anal. for C_38_H_30_Cl_2_N_4_O_6_S_4_ calcd; C, 54.47; H, 3.61; N, 6.69; S, 15.31. Found: C, 54.17; H, 3.31; N, 6.39; S, 15.01.

#### 3.7.4. 1-(3,4-Dimethyl-5-2-(2-(p-tolyl)hydrazono)-2-tosylacetyl)thieno[2,3-*b*]thiophen-2-yl)-2-(2- (*p*-tolyl)hydrazono)-2-tosylethanone (**10c**)

**10b** was prepared according to (GP4), dark yellow needles crystals; yield (77%); m.p. 300–302 °C; IR *ν*_max_ (KBr) 3356 (NH), 1652 (C=O), 1582 (C=N) cm^−1; 1^H-NMR (400 MHz, DMSO-*d*_6_) (ppm): 2.05, 2.10, 2.86 (s, 18H, CH_3_), 6.67 (d, 2H, *J* = 8.8 Hz, ArH’s), 6.81 (d, 2H, *J* = 8.8 Hz, ArH’s), 7.62 (d, 2H, *J* = 7.3 Hz, ArH’s), 7.79 (d, 2H, *J* = 7.3 Hz, ArH’s), 9.52 (br, 2H, NH); ^13^C-NMR: *δ* 16.0, 24.2, 24.1, 120.1, 120.9, 122.4, 123.9, 127.8, 128.2, 134.1, 137.6, 142.0, 143.8, 146.6, 152.9, 163.3, 188.0; MS *m/z* (%): 796 (M^+^, 28); Anal. for C_40_H_36_N_4_O_6_S_4_ calcd; C, 60.28; H, 4.55; N, 7.03; S, 16.09. Found: C, 60.08; H, 4.25; N, 7.33; S, 16.29.

## 4. Conclusions

In conclusion, the present investigation describes an efficient method for access toward novel *bis*-heterocycles containing biologically active moieties. We believe that these new series of symmetrical *bis*-hetrocycles may exhibit potentially diverse useful applications in the field of medicinal chemistry.
